# Brain processing of pain in patients with unresponsive wakefulness syndrome

**DOI:** 10.1002/brb3.110

**Published:** 2013-01-28

**Authors:** Alexandra Markl, Tao Yu, Dominik Vogel, Friedemann Müller, Boris Kotchoubey, Simone Lang

**Affiliations:** 1 Schön Klinik Bad AiblingKolbermoorer Straβe 72, 83043 Bad Aibling, Germany; 2 Institute of Medical Psychology and Behavioral Neurobiology, Eberhard-Karls-University TübingenGartenstraβe 29, 72074 Tübingen, Germany; 3 Institute of Psychology, Department of Clinical Psychology and Psychotherapy, Ruprecht-Karls-University Heidelberg, Hauptstrasse 47-5169117 Heidelberg, Germany

**Keywords:** Functional magnetic resonance imaging, pain, unresponsive wakefulness syndrome

## Abstract

By definition, patients with unresponsive wakefulness syndrome (UWS) do not experience pain, but it is still not completely understood how far their brain can process noxious stimuli. The few positron emission tomography studies that have examined pain processing did not yield a clear and consistent result. We performed an functional magnetic resonance imaging scan in 30 UWS patients of nontraumatic etiology and 15 age- and sex-matched healthy control participants (HC). In a block design, noxious electrical stimuli were presented at the patients' left index finger, alternating with a resting baseline condition. Sixteen of the UWS patients (53%) showed neural activation in at least one subsystem of the pain-processing network. More specifically, 15 UWS patients (50%) showed responses in the sensory-discriminative pain network, 30% in the affective pain network. The data indicate that some patients completely fulfilling the clinical UWS criteria have the neural substrates of noxious stimulation processing, which resemble that in control individuals. We therefore suppose that at least some of these patients can experience pain.

## Introduction

Pain is defined as an unpleasant sensory and emotional experience, associated with real or potential tissue damage (Merskey and Bogduk 1994) and including both physical (i.e., nociception which means the detection of pain-producing stimuli by primary sensory neurons) and affective aspects (i.e. suffering) (Kupers et al. 2005).

In individuals with preserved consciousness, both positron emission tomography (PET) and functional magnetic resonance imaging (fMRI) reveal a well-formed network of interrelated brain regions responding to pain stimuli, the so-called pain matrix. The pain matrix entails two main subsystems (Brooks and Tracey 2005): the lateral neuronal network that encodes sensory-discriminative information consists of the primary (S1) and the secondary somatosensory (S2) cortex, the lateral thalamus, and the posterior insula (Mutschler et al. 2011). The medial network that encodes affective-cognitive information consists of the anterior insula, the anterior cingulate cortex (ACC), and the prefrontal cortex (Wiech et al. 2001; Medford and Critchley 2010). Although the cerebellum does actually not belong to the so-called pain matrix, it is known that it plays a role in processing aversive stimuli including pain (Moulton et al. 2011). Therefore, it can be counted to the sensory-discriminative part of the pain-processing network. Furthermore, motor-related areas (e.g., the striatum, cerebellum, and the supplementary motor area) are involved in pain perception and processing (Barceló et al. 2012).

The unresponsive wakefulness syndrome (UWS; former vegetative state) (Laureys et al. 2010) is a state of preserved wakefulness, but absent voluntary behavioral signs and subjective experiences (Jennett and Plum 1972; Multi Society Task Force on PVS 1994). Because pain is regarded as subjective experience (Merskey and Bogduk 1994), and UWS is defined as the complete lack of any subjective phenomena (Jennett and Plum 1972), it follows that the patients cannot feel pain. This assumption may have far-going consequences, from a small surgery performed without anesthesia up to serious ethical and legal decisions, even the end-of-life decisions in such patients (Demertzi et al. 2012). Notwithstanding these possibly critical consequences, the assumption of UWS patients' inability to experience nociceptive stimuli and suffer from pain remains unproven to date. The fact that the rate of misdiagnosis of UWS is about 40% (Childs et al. 1993; Andrews et al. 1996; Schnakers et al. 2009) may indicate that a number of patients fulfilling all clinical criteria can nevertheless possess components of awareness.

Several attempts to clarify this issue have been undertaken using PET. Laureys et al. (2002) used ^15^O-radiolabeled water PET to study cortical processing of noxious stimulation of the median nerve and found significant brain activations in the midbrain, contralateral thalamus, and S1 in each of the 15 examined UWS patients. However, the activated primary cortex was functionally disconnected from secondary, higher order integrative brain regions. Similar results were obtained in two other PET studies with a medium-size sample (Laureys et al. 2002; Boly et al. 2008). Contrary to these studies, Kassubek et al. (2003) found in seven UWS patients that a broad pain-related cerebral network, including higher order associative areas, can remain active even in long-term UWS patients.

It should be noted that there has been no fMRI study of noxious processing in UWS. Although a comparative analysis of advantages and disadvantages of PET versus fMRI (Bruckner and Logan 2001) would go beyond the frame of this article, it can be said that generally fMRI has a higher spatial and temporal resolution. This study aimed at using fMRI for the first time to investigate noxious processing in a larger sample of 30 nontraumatic UWS patients.

## Methods

### Participants

During a sample period of 22 months, 50 patients with UWS were screened. Twenty of them had to be excluded due to medical or other reasons (magnetic resonance imaging [MRI] or medical exclusion criteria, *n* = 6; palliative care or death, *n* = 5; discharged from hospital, *n* = 3; refusal of informed consent, *n* = 6). Thirty UWS patients fulfilling the inclusion criteria underwent the fMRI examination (16 males, mean age 48.4 ± 15.5 years, range 16–72) as well as 15 healthy participants (eight males, mean age 42.4 ± 11.8 years) (Table 1).

**Table 1:** Clinical characteristics of patients

No	Scanner	Sex	Age	Etiology	Time interval in months	CRS-R	CRS-R subscores: auditive–visual–motor–oromotor–communication–arousal	Atrophy
1	1.5	F	16	Anoxic	20	5	1–1–0–1–0–2	2
2	1.5	M	36	Anoxic	3	6	1–0–2–1–0–2	3
3	1.5	F	64	Anoxic	104	6	1–1–1–1–0–2	4
4	1.5	F	69	Hemorrhage	39	5	1–1–1–1–0–1	4
5	1.5	F	38	Anoxic	25	5	1–1–1–1–0–1	3
6	1.5	F	52	Anoxic	60	8	2–1–2–1–0–2	4
7	1.5	F	71	Anoxic	2	5	1–0–1–1–0–2	2
8	1.5	M	36	Anoxic	9	6	1–0–2–1–0–2	3
9	1.5	F	56	Hemorrhage	33	6	1–1–1–1–0–2	3
10	1.5	M	44	Hemorrhage	23	4	1–0–1–1–0–1	4
11	1.5	M	29	Anoxic	34	6	1–1–1–1–0–2	3
12	1.5	F	63	Anoxic	2	5	1–0–2–1–0–1	2
13	1.5	M	19	Anoxic	4	7	1–0–2–2–0–2	2
14	1.5	M	40	Encephalopathy	3	7	1–0–2–2–0–2	2
15	1.5	M	36	Anoxic	50	4	1–0–1–0–0–2	4
16	1.5	F	62	Hemorrhage	4	6	1–1–2–1–0–1	2
17	1.5	F	30	Anoxic	1	5	1–1–0–1–0–2	2
18	1.5	M	57	Anoxic	57	5	0–0–2–1–0–2	4
19	1.5	M	44	Anoxic	50	7	1–1–2–1–0–2	3
20	1.5	F	62	Anoxic	66	7	2–1–1–1–0–2	4
21	1.5	M	25	Anoxic	3	6	1–0–2–1–0–2	2
22	1.5	M	51	Anoxic	1	5	1–0–1–1–0–2	1
23	3	M	64	Anoxic	111	–^1^	–^1^	4
24	3	M	55	Anoxic	80	6	1–1–1–1–0–2	4
25	3	M	47	Anoxic	64	5	1–0–1–1–0–2	4
26	3	M	75	Anoxic	20	–^1^	–^1^	3
27	3	F	53	Anoxic	84	–^1^	–^1^	4
28	3	F	54	Anoxic	93	5	1–0–1–1–0–2	4
29	3	F	45	Anoxic	287	2	0–0–1–0–0–1	4
30	3	M	59	Anoxic	88	–^1^	–^1^	4

CRS-R, Coma Recovery Scale – Revised.

^1^CRS-R examination was not possible.

All patients were of nontraumatic etiology, including hypoxic encephalopathy (*n* = 25), subarachnoid or intracerebral hemorrhage (*n* = 4) and encephalitis (*n* = 1). Patients' morphologic information provided by T1-weighted scans was assessed using a scale developed by Galton et al. (2001) and Bekinschtein et al. (2011) (from 0 = no atrophy to 4 = very severe atrophy). The degree of atrophy was evaluated by three experienced raters who were blind concerning the identity of patients. The mean degree of atrophy was 3.1 (±0.9) and the value of the Coma Recovery Scale was on average 5.4 (±1.4).

The diagnosis was made on the basis of careful, repeated clinical examination including the Coma Recovery Scale – Revised (CRS-R) (Giacino et al. 2004).

Twenty-six patients underwent a CRS-R examination within the first week of their stay and then every 2 weeks. Within the week before the MR scan, another CRS-R score was determined, which went into our analysis.

In four patients, the standardized examination according to the CRS-R was not possible. They had to be transported over a long distance and were directly brought to the scanning center. All of them were chronic patients. Their diagnoses have been verified by their attending physicians.

Exclusion criteria for healthy participants were the history of head trauma, neurological diseases, or any chronic illness. Exclusion criteria for all participants were any contraindication to fMRI. The participants' legal guardians gave written informed consent. The study was approved by the ethical committee of the University of Tuebingen and conducted in accordance with the Declaration of Helsinki.

### Experimental procedure

An alternating block design (three noxious stimulation blocks, three baseline blocks) was performed. Each block consisted either of 60 noxious stimuli (1/sec) or a 60-sec baseline rest interval. The nociceptive experience was elicited by an electrical stimulus (5 mA, 200 msec) at the left index finger using the DS7A HV Constant Current Stimulator from Digitimer. The HC group evaluated the electrical stimuli as moderately painful (mean 3.93; SD = 1.28) on a visual analogue scale (VAS) from 0 (no pain) to 10 (worst pain imaginable). The moderate pain stimulation was used for ethical reasons. Another group of 16 healthy individuals (seven males, mean age 25.7 [SD = 4.41]), who did not participate in the fMRI experiment, additionally evaluated the valence (mean 7.81, SD = 0.91 on the scale from 1 = very pleasant to 9 = very unpleasant) and arousal (mean 7.31, SD = 1.54 on the scale from 1 to 9) of the same stimuli.

The examination was always accompanied by a physician. The patient's vital signs (heart rate, oxygen saturation) were monitored continuously.

### Image acquisition and statistical analysis

Blood oxygenation level-dependent (BOLD) images were obtained at two imaging centers (Bad Aibling and Tuebingen, Germany) in order to avoid unnecessary patient transportation. In Bad Aibling, where 22 patients were examined, data were collected using a 1.5 Tesla MRI scanner (TIM Symphony; Siemens Medical Systems, Erlangen, Germany) system equipped with a 12-channel head coil. Changes in BOLD T2*-weighted MR signal were measured using a gradient echo-planar imaging (EPI) sequence (TR = 3410 msec, TE = 50 msec, FoV = 192 mm, flip angle = 90°, 64 × 64 matrix, 36 slices covering the whole brain, slice thickness 3.0 mm, no gap, voxel size 3 × 3 × 3 mm). A T1-weighted anatomical image was additionally acquired for each subject to allow anatomical localization (TR = 2300 msec, TE = 2.98 msec, 160 slices, voxel size 1.0 × 1.0 × 1.1 mm). In Tuebingen, imaging was performed on a 3 T Siemens Trio scanner. After a T2*-weighted acquisition (TR = 2380 msec, echo time = 25 msec, FoV = 210 mm, flip angle = 90°, 64 × 64 matrix, 40 slices covering the whole brain, slice thickness 3 mm, no gap, voxel size 3.3 × 3.3 × 3.0 mm), anatomical images were obtained using the MP-RAGE sequence (repetition time = 2300 msec, echo time = 2.98 msec, 160 slices, slice thickness = 1 mm, voxel size 1.0 × 1.0 × 1.1 mm).

Magnetic resonance imaging scans of the 15 healthy subjects were acquired in Bad Aibling using the above-mentioned 1.5 T Siemens Symphony MR Scanner and the same imaging parameters.

Image processing and statistical analysis were conducted using Statistical Parametric Mapping (Friston et al. 1995) version 8 (Wellcome Department of Cognitive Neurology, London, UK; http://www.fil.ion.ucl.ac.uk/spm/software/spm8/). Preprocessing included realignment, coregistration, segmentation, and spatial normalization (template of Montreal Neurological Institute [MNI]). Then, a Gaussian filter of 8-mm full width at half maximum was applied to smooth the data spatially.

For the statistical analysis of regional differences in brain activation, painful stimulation and resting condition were input into the categorical general linear model design at the subject level (Friston et al. 1995). Contrasts between pain and baseline conditions were computed for each subject. In addition, main effects were computed using one-sample *t-*tests for each group (UWS, HC) separately. The probability threshold was set at *P* < 0.05, corrected for family-wise errors (FWE) for whole-brain analysis.

In addition, region of interest (ROI) analyses were performed for pain-related brain areas on the individual level, such as the ACC, insula, S1, S2, thalamus, and cerebellum using automated anatomical labeling masks (Tzourio-Mazoyer et al. 2002) and the WFU Pickatlas (Maldjian et al. 2003). ROI analyses were applied in HCs and patients. The ROIs were superimposed onto each patient's T1 image with manual adjustments to those anatomical landmarks if necessary (Bekinschtein et al. 2011). A significance level of *P* < 0.05 (FWE corrected) was used.

For comparison between UWS and HC, several chi-squared tests were applied. Their significance was corrected by the number of the tests using the Bonferroni–Holm correction procedure (Holm 1979).

## Results

### Healthy subjects

**Figure 1:**
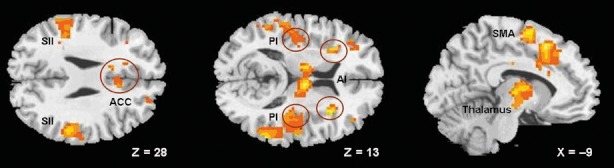
Significant activation observed in healthy subjects in response to the painful stimulation (Pain) versus rest (No pain). The height threshold was *P* < 0.001 (uncorrected) for illustrating.

As can be seen in Table 2 and Figure 1, in the healthy group, noxious stimuli significantly activated the S1 and S2, the anterior cingulate gyrus (ACC), the inferior frontal gyrus, the insula, the thalamus, and the cerebellum.

**Table 2:** Brain regions activated by pain stimulation in healthy control group

				Peak in MNI			
					
Region	L/R	BA	Cluster size (voxels)	*x*	*y*	*z*	*z*-score
SMA/ACC	L	6/24	704	−6	17	52	5.36
Thalamus	L		285	−15	2	1	5.00
Insula	R	13	705	45	−28	19	4.63
Inferior parietal gyrus/postcentral gyrus	R	40/2	–^1^	51	−31	25	4.45
Inferior parietal lobule	L	40	464	−42	−34	22	4.21
Insula/postcentral gyrus	L	13/2	–^1^	−42	−16	7	4.21
Precentral gyrus	R	6	176	39	−10	55	4.05
Inferior frontal gyrus	R	44	54	51	5	16	4.08

Clusters identified with a threshold of *P* < 0.05 family-wise error corrected for multiple comparisons.

L, left hemisphere; R, right hemisphere; BA, number of Brodman area; SMA, supplementary motor area; ACC, anterior cingulate cortex; MNI, Montreal Neurological Institute atlas.

^1^No information on coordinates and/or cluster size because regions belong to higher clusters.

The data presented in Table 3 indicate that all HC subjects showed a significant activation in the S1 and higher order brain structures (insula, ACC, S2, and cerebellum). Nine HC subjects (60%) exhibited significant activation not only in the sensory but also in the affective part of the pain system (ACC, anterior insula). Activation in the lower order brain structures (S1 and thalamus) was found in 12 (80%) HC subjects.

**Table 3:** Individual results of the pain-minus-rest contrast for each of the selected region of interests in healthy controls

Subject number	Sex	Age	ACC	AI	S2	S1	Thalamus	PI	Cerebellum	VAS score
1	F	52	−	−	+	+	−	−	+	2.5
2	F	29	−	+	+	+	+	+	+	4
3	M	46	−	−	+	−	+	−	+	4.5
4	M	29	+	+	+	+	+	+	+	3
5	F	31	+	+	+	+	+	+	+	3
6	F	35	+	+	+	−	−	+	−	4
7	M	32	+	+	+	+	+	+	−	4
8	M	62	−	−	+	−	−	+	−	7
9	F	47	−	−	−	+	−	+	−	1.5
10	M	52	−	+	+	+	−	+	−	5
11	F	58	−	−	+	+	−	−	−	3
12	M	48	+	+	+	+	−	−	−	2.5
13	F	28	+	+	+	+	+	+	+	3
14	M	33	−	+	+	+	−	+	+	3
15	M	54	−	−	+	−	−	−	−	6

Significant brain activation (region of interests) identified with a threshold of *P* < 0.05 family-wise error corrected for multiple comparisons.

Sex (F, female; M, male); ACC, anterior cingulate cortex; AI, anterior insula; S2, secondary somatosensory cortex; S1, primary somatosensory cortex; PI, posterior insula; VAS, visual analogue scale for pain rating (from 0 = no pain at all, to 10 = worst pain imaginable); +, significantly positive blood oxygenation level-dependent signal in the pain-stimulation condition compared with baseline condition; −, no significant response.

### UWS patients

**Figure 2:**
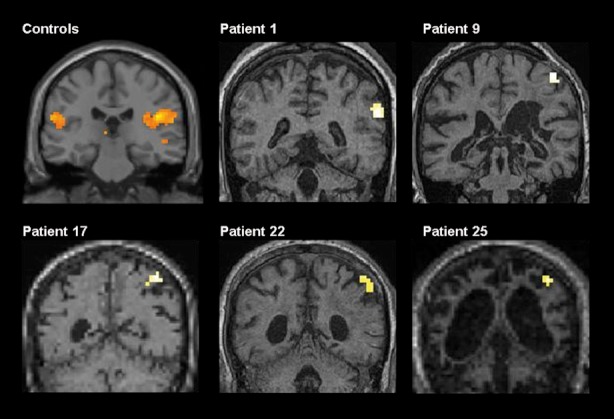
Significant individual brain responses in the secondary somatosensory cortex.

As can be seen in Table 4 and Figure 2, 15 UWS patients (50%) exhibited significant activations in the sensory part of the pain matrix and/or the cerebellum, nine (30%) UWS patients exhibited significant activations in the affective part of the pain matrix (ACC and/or anterior insula), and in eight (26.7%) UWS patients both sensory (including cerebellum) and affective components were activated. Activation in the higher order structures was found in 15 (50%) UWS patients and lower order structures were activated in four patients (13.3%).

**Table 4:** Individual results of the pain-minus-rest contrast for each of the selected region of interests in unresponsive wakefulness syndrome patients

Patient number	ACC	AI	S2	S1	Thalamus	PI	Cerebellum
1	−	−	+	+	−	+	+
2	−	−	−	−	−	−	−
3	+	−	−	−	−	−	+
4	−	−	−	−	−	−	−
5	+	−	−	−	−	−	−
6	−	−	−	−	−	−	−
7	+	−	−	−	−	−	+
8	+	+	−	−	−	−	+
9	−	−	+	−	−	+	−
10	−	+	−	−	−	−	+
11	−	+	−	−	+	−	+
12	−	−	−	−	−	−	+
13	−	−	−	−	−	−	−
14	−	−	−	−	−	−	−
15	−	−	−	−	−	−	+
16	−	−	−	+	−	−	−
17	−	+	+	+	−	−	+
18	−	−	−	−	−	−	−
19	−	−	−	−	−	−	−
20	−	−	−	−	−	−	−
21	−	−	−	−	−	−	−
22	+	−	+	−	−	+	−
23	−	−	−	−	−	−	−
24	−	−	−	−	−	−	+
25	−	+	+	−	−	−	−
26	−	−	−	−	−	−	−
27	−	−	−	−	−	−	−
28	−	−	−	−	−	−	+
29	−	−	−	−	−	−	−
30	−	−	−	−	−	−	−

Significant brain activation (region of interests) identified with a threshold of *P* < 0.05 family-wise error corrected for multiple comparisons.

ACC, anterior cingulate cortex; AI, anterior insula; S2, secondary somatosensory cortex; S1, primary somatosensory cortex; PI, posterior insula; +, significantly positive blood oxygenation level-dependent signal in the pain-stimulation condition compared with baseline condition; −, significant response.

Acute and subacute patients (<3 months in UWS; *n* = 4) tended to demonstrate significant activations in the sensory-discriminative network more often than chronic patients (≥3 months in UWS; *n* = 26: *P* = 0.05, Fisher exact test, one tailed). The same trend for the affective network only approached significance (*P* = 0.069).

The CRS-R motor subscore, which reflects motor reaction to nociceptive stimulation (0 = no reaction/flaccid; 1 = abnormal posturing; 2 = flexion withdrawal) was significantly negative related with a general absence/presence of brain activation in the regions of the pain network (Spearman ρ = −0.52; *P* = 0.006) and with the number of activated regions (Spearman ρ = −0.60; *P* = 0.001). None of the patients showed a CRS-R motor subscore higher than 2 (else the diagnosis would be different from UWS).

### Group differences regarding individual brain activation

Compared with UWS patients, HC subjects showed significantly more frequent activation in the sensory and the affective part of the pain matrix (χ^2^ = 11.25, *P* < 0.001; χ^2^ = 7.61, *P* = 0.010, for the sensory and affective subsystems, respectively) and in the higher and lower order structures (χ^2^ = 11.25, *P* < 0.001; χ^2^ = 19.40, *P* < 0.001, respectively).

Looking at the individual brain areas demonstrated that the anterior insula (χ^2^ = 8.76, *P* = 0.009), the S2 (χ^2^ = 24.09, *P* = 0.007), the S1 (χ^2^ = 18.72, *P* = 0.006), the thalamus (χ^2^ = 10.24, *P* = 0.004), and the posterior insula (χ^2^ = 15.63, *P* = 0.005) were significantly more frequently activated in HC subjects than UWS patients. No significant group difference was found in the cerebellum (χ^2^ = 0.42, *P* = 0.52) and in the ACC (χ^2^ = 2.95, *P* = 0.172).

## Discussion

This is the first study investigating the individual brain activations elicited by noxious stimuli in a large homogenous sample of UWS patients using fMRI.

### Healthy controls

Noxious stimulation significantly activated brain areas previously described in brain-imaging studies of pain using electrical and other noxious stimulation (Price 2002; Apkarian et al. 2005; Mutschler et al. 2011). Thus, the present data successfully replicated the results of the previous pain-imaging studies. On the individual level, all healthy subjects showed brain activation in the sensory-discriminative subsystem of the pain matrix and the higher order brain structures (insula, ACC, S2, and cerebellum) during painful stimulation. Twelve individuals (80%) significantly activated lower order brain areas, such as S1 and thalamus. However, only nine individuals (60%) exhibited activation in the affective subsystem (ACC, anterior insula).

One reason that six HCs did not show significant neural responses in the affective subsystem of the pain matrix could be the relative mild intensity of the pain stimulation (5 mA), although the average rating of the pain intensity was 3.93 (SD = 1.28) on a VAS (from 0 = no pain at all to 10 = worst pain imaginable). Another more probable explanation from our point of view is the age of the HC patients: those healthy participants who do not show any activation in the affective part of the pain matrix were 18 years older on average. The correlation between age and BOLD signal in HC is 0.7, whereas there is no correlation between VAS score and BOLD in HC, and between age and BOLD signal in UWS patients. It is known that the subjective threshold of pain experience increases with age (Gibson and Helme 2001).

### Unresponsive wakefulness state

The inability to experience pain (or any other subjective feeling) is a central part of the definition of the UWS. Despite this definition, not only patients' relatives but also many health professionals believe that UWS patients can perceive pain. Demertzi et al. (2009) asked 2059 medical and paramedical professionals in Europe about their beliefs concerning the vegetative or minimally conscious states. Of the paramedical caregivers and medical doctors, 68% and 56%, respectively, believed that a UWS patient can feel pain.

In several studies using PET, pain-related activations in UWS patients were observed exclusively in the primary sensory part of the pain matrix, indicating that higher order processing of noxious stimuli was lacking (e.g. Boly et al. 2005, 2008). In a smaller study of Kassubek et al. (2003), in contrast, activations of the sensory-discriminative and affective parts of the pain network were obtained in each of the examined seven UWS patients.

The present data indicate clear individual differences in pain responsiveness of UWS patients. While no pain-related activation was found in 14 patients, the others did respond to noxious stimuli. Fifteen UWS patients showed activation in the sensory-discriminative part of the pain matrix and nine patients (30%) showed even affective pain-related responses. In 15 patients, higher order pain control areas (ACC, anterior insula, S2) were also involved. Moreover, about one-third of the UWS patients had pain-related responses in both, the sensory and affective parts of the pain matrix, thus replicating the PET findings of Kassubek et al. (2003) at least for a subgroup of UWS patients. In our study, a trend to more frequent activations of the pain matrix in (sub)acute UWS patients as compared with chronic UWS patients was observed.

The somatosensory cortex receives noxious input from the thalamus and contains neurons that code spatial, temporal, and intensity of noxious somatosensory stimuli (Derbyshire 2000; Price 2002), characteristics that constitute the sensory-discriminative dimension of pain processing. The ACC and the insular cortex are considered to be brain areas of the classical limbic system and are involved in the processing of the affective-motivational dimension of pain (Price 2002). The insula has been implicated in pain sensation and comprise the only cortical areas in which direct electrical stimulation produces a perception of pain (Ostrowsky et al. 2002; Frot and Mauguiere 2003). Especially, the anterior insula is related to interoceptive awareness (Craig 2008), emotional salience, awareness of subjective feeling, and bodily arousal states (Craig 2002). This structure has also been reported in self-referential tasks in the emotional domain (Modinos et al. 2009).

The CRS-R motor subscore was significantly and inversely correlated with a general absence/presence of brain activation in the pain network and with the number of activated regions. At first sight, this result is surprising, as one might have expected a positive correlation between brain activation in the pain matrix and the clinical reaction to noxious stimuli. However, absent motor reaction to pain stimulation does not necessarily mean the absence of pain perception. Perception of pain, more precisely the sensory afference, and the visible motor reaction, thus the motor efference, are two different and independent pathways and can be impaired separately from each other. In fact, our findings emphasize that the clinical examination of UWS patients is difficult and might miss some patients who are actually more conscious than they seem (Childs et al. 1993; Andrews et al. 1996; Schnakers et al. 2009). This fact stresses the necessity for new methods, like functional imaging, to examine patients with disorders of consciousness objectively.

Of course, diagnosis of consciousness remains a philosophical problem, not just a neurological one. An increase of brain activity in some brain areas cannot strongly prove the presence of subjective experience. However, given pain-related changes in such brain structures as anterior insula and ACC, which are related to emotional awareness and autonomic regulation of pain (Vogt 2005), we find it risky to still argue that the respective patients are unable to feel pain. Note that among these were also chronic patients (several years after the incident) with a very severe hypoxic brain injury.

### Limitations

By definition, subjective threshold of pain perception and pain tolerance cannot be obtained in UWS patients. Therefore, and primarily for ethical reasons, rather moderate noxious stimuli were applied in the present experiment. We cannot rule out that they were weaker than in the comparable PET studies, which would partially explain the differences in results.

Moreover, to avoid long, uncomfortable, and exhausting transportation, the patients were examined at two different imaging centers with different scanners. Although the results obtained with the 1.5 T and the 3 T scanners are not substantially different, a replication study in which all patients are measured with the same scanner is desirable.

Furthermore, Boyle et al. (2006) demonstrated that MRI scanner noise significantly reduces unpleasantness ratings of pain stimulation. Although healthy individuals evaluated the presented stimuli as highly arousing and rather unpleasant, it should be taken into account that the evaluations were done immediately outside MRI scan (i.e., without noise). The difference in the physical environment should, therefore, also be considered when discussing neuroimaging studies on human pain perception.

Importantly, these limitations may be supposed to decrease, rather than enhance, the pain responsiveness of the patients. The fact that the present experiment was strongly biased toward false-negative findings underscores the importance of positive ones. If stimuli of such low intensity, perhaps additionally masked by the scanner noise, activated large portions of the brain pain matrix in one third of our UWS sample, one can suppose that in a real and severe pain event (e.g., toothache) the brain activation might be even more pronounced. From a practical point of view, therefore, a conclusion from the present data may be drawn that the medical staff should carefully examine UWS patients for any clinical sign or potential source of pain and treat them appropriately, assuming in the case of doubt that pain is subjectively experienced unless strong evidence for the opposite is obtained.

The brain responses to pain were contrasted to the rest condition only. As a next step, it would be interesting to compare brain responses to painful and nonpainful (e.g., touch) somatosensory stimuli. The present, rather plain design was selected to provide the comparability with the previous PET studies of UWS patients, in which the same design was employed.

## Conclusions

This is the first fMRI study on pain processing in a larger group of patients in UWS. Significant indications of pain processing were found in at least half of UWS patients, and about one-third UWS patients showed even activations in both sensory and affective pain networks. The findings stress the need for elaborated pain management in patients with disorders of consciousness.

## References

[b1] Andrews K, Murphy L, Munday R, Littlewood C (1996). Misdiagnosis of the vegetative state: retrospective study in a rehabilitation unit. BMJ.

[b2] Apkarian AV, Bushnell MC, Treede RD, Zubieta JK (2005). Human brain mechanisms of pain perception and regulation in health and disease. Eur. J. Pain.

[b3] Barceló AC, Filippini B, Bazo JH (2012). The striatum and pain modulation. Cell. Mol. Neurobiol.

[b4] Bekinschtein TA, Manes FF, Villarreal M, Owen AM, Della-Maggiore V (2011). Functional imaging reveals movement preparatory activity in the vegetative state. Front Hum. Neurosci.

[b5] Boly M, Faymonville ME, Peigneux P, Lambermont B, Damas F, Luxen A (2005). Cerebral processing of auditory and noxious stimuli in severely brain injured patients: differences between VS and MCS. Neuropsychol. Rehabil.

[b6] Boly M, Faymonville ME, Schnakers C, Peigneux P, Lambermont B, Phillips C (2008). Perception of pain in the minimally conscious state with PET activation: an observational study. Lancet Neurol.

[b7] Boyle Y, Bentley DE, Watson A, Jones AK (2006). Acoustic noise in functional magnetic resonance imaging reduces pain unpleasantness ratings. NeuroImage.

[b8] Brooks J, Tracey I (2005). From nociception to pain perception: imaging the spinal and supraspinal pathways. J. Anat.

[b9] Bruckner RL, Logan JM (2001). Handbook of functional neuroimaging of cognition.

[b10] Childs NL, Mercer WN, Childs HW (1993). Accuracy of diagnosis of persistent vegetative state. Neurology.

[b11] Craig AD (2002). How do you feel? Interoception: the sense of the physiological condition of the body. Nat. Rev. Neurosci.

[b12] Craig AD, Lewis M, Feldman-Barrett L (2008). Interoception and emotion: a neuroanatomical perspective. Handbook of emotions.

[b13] Demertzi A, Schnakers C, Ledoux D, Chatelle C, Bruno MA, Vanhaudenhuyse A (2009). Different beliefs about pain perception in the vegetative and minimally conscious state: a European survey of medical and paramedical professionals. Prog. Brain Res.

[b14] Demertzi A, Racine E, Bruno MA, Ledoux D, Gosseries O, Vanhaudenhuyse A (2012). Pain perception in disorders of consciousness: neuroscience, clinical care and ethics in dialogue. Neuroethics.

[b41] Derbyshire SWG (2000). Exploring the pain neuromatrix. Curr. Rev. Pain.

[b15] Friston KJ, Holmes AP, Worsley KJ, Poline JB, Frith C, Frackowiak RSJ (1995). Statistical parametric maps in functional imaging: a general linear approach. Hum. Brain Mapp.

[b16] Frot M, Mauguiere F (2003). Dual representation of pain in the operculoinsular cortex in humans. Brain.

[b17] Galton CJ, Gomez-Anson B, Antoun N, Scheltens P, Patterson K, Graves M (2001). Temporal lobe rating scale: application to Alzheimer's disease and frontotemporal dementia. J. Neurol. Neurosurg. Psychiatry.

[b18] Giacino J, Kalmar K, Whyte J (2004). The JFK Coma Recovery Scale-Revised: measurement characteristics and diagnostic utility. Arch. Phys. Med. Rehabil.

[b19] Gibson SJ, Helme RD (2001). Age related differences in pain perception and report. Clin. Geriatr. Med.

[b20] Holm S (1979). A simple sequentially rejective multiple test procedure. Scand. J. Stat.

[b21] Jennett B, Plum F (1972). Persistent vegetative state after brain damage: a syndrome in search of a name. Lancet.

[b22] Kassubek J, Jeungling FD, Els T, Spreer J, Herpers M, Krause T (2003). Activation of a residual cortical network during painful stimulation in long-term postanoxic vegetative state: a 15-O-H2O PET study. J. Neurol. Sci.

[b23] Kupers R, Faymonville ME, Laureys S (2005). The cognitive modulation of pain: hypnosis- and placebo-induced analgesia. Prog. Brain Res.

[b24] Laureys S, Faymonville ME, Peigneux P, Damas P, Lambermont B, Del Fiore G (2002). Cortical processing of noxious somatosensory stimuli in the persistent vegetative state. NeuroImage.

[b25] Laureys S, Celesia G, Cohadon F, Lavrijsen J, Léon-Carrrion J, Sannita WG (2010). Unresponsive wakefulness syndrome: a new name for the vegetative state or apallic syndrome. BMC Med.

[b26] Maldjian JA, Laurienti PJ, Kraft RA, Burdette JH (2003). An automated method for neuroanaomic and cytoarchitectonic atlas-based interrogation of fMRI data sets. NeuroImage.

[b27] Medford N, Critchley HD (2010). Conjoint activity of anterior insular and anterior cingulate cortex: awareness and response. Brain Struct. Funct.

[b28] Merskey H, Bogduk N (1994). Classification of chronic pain.

[b29] Modinos G, Ormel J, Aleman A (2009). Activation of anterior insula during self-reflection. PLoS ONE.

[b30] Moulton EA, Alman I, Pendse G, Schmahmann J, Becerra L, Borsook D (2011). Aversion-related circuitry in the cerebellum: responses to noxious heat and unpleasant images. J. Neurosci.

[b31] Mutschler I, Wankerl J, Seifritz E, Ball T (2011). The role of the human insular cortex in pain processing. Eur. Psychiatry.

[b32] Ostrowsky K, Magnin M, Ryvlin P, Isnard J, Guenot M, Mauguière F (2002). Representation of pain and somatic sensation in the human insula: a study of responses to direct electrical cortical stimulation. Cereb. Cortex.

[b33] Price DD (2002). Central neural mechanisms that interrelate sensory and affective dimensions of pain. Mol. Interv.

[b35] Schnakers C, Vanhaudenhuyse A, Giacino J, Ventura M, Boly M, Majerus S (2009). Diagnostic accuracy of the vegetative and minimally conscious state: clinical consensus versus standardized neurobehavioral assessment. BMC Neurol.

[b37] The Multi Society Task Force on PVS (1994). Medical aspects of the persistent vegetative state. N. Engl. J. Med.

[b38] Tzourio-Mazoyer N, Landeau B, Papathanassiou D, Crivello F, Etard O, Delcroix N (2002). Automated anatomical labeling of activations in SPM using a macroscopic anatomical parcellation of the MNI MRI single-subject brain. NeuroImage.

[b39] Vogt BA (2005). Pain and emotion interactions in subregions of the cingulate gyrus. Nat. Rev. Neurosci.

[b40] Wiech K, Preissl H, Birbaumer N (2001). Neural networks and pain processing. New insights from imaging techniques. Anaesthesist.

